# Supplementary material for: Estimation of Excess All-Cause Mortality Due to COVID-19 in Thailand
Source: Trop Med Infect Dis. 2022 Jun 24;7(7):116. doi: 10.3390/tropicalmed7070116 (PMC9322618; doi:10.3390/tropicalmed7070116)
Supplement: Supplementary file 1 [file tropicalmed-07-00116-s001.zip › tropicalmed-1769808-supplementary.pdf]

Supplementary Materials

Estimation of Excess All-Cause Mortality Due to COVID-19 in Thailand

Chaiwat Wilasang [1], Charin Modchang [1,2,3], Thanchanok Lincharoen [1] and Sudarat Chadsuthi [4,*]

[1] Biophysics Group, Department of Physics, Faculty of Science, Mahidol University, Bangkok 10400, Thailand; chaiwat.wil@student.mahidol.ac.th (C.W.); charin.mod@mahidol.edu (C.M.); thanchanok.lin@student.mahidol.edu (T.L.)
[2] Centre of Excellence in Mathematics, MHESI, Bangkok 10400, Thailand
[3] Thailand Center of Excellence in Physics, Ministry of Higher Education, Science, Research and Innovation, 328 Si Ayutthaya Road, Bangkok 10400, Thailand
[4] Department of Physics, Research Center for Academic Excellence in Applied Physics, Faculty of Science, Naresuan University, Phitsanulok 65000, Thailand
* Correspondence: sudaratc@nu.ac.th

Deaths due to COVID-19

The number of daily COVID-19 deaths in Thailand (Figure S1) was retrieved from the Center for Systems Science and Engineering (CSSE) at Johns Hopkins University [1].

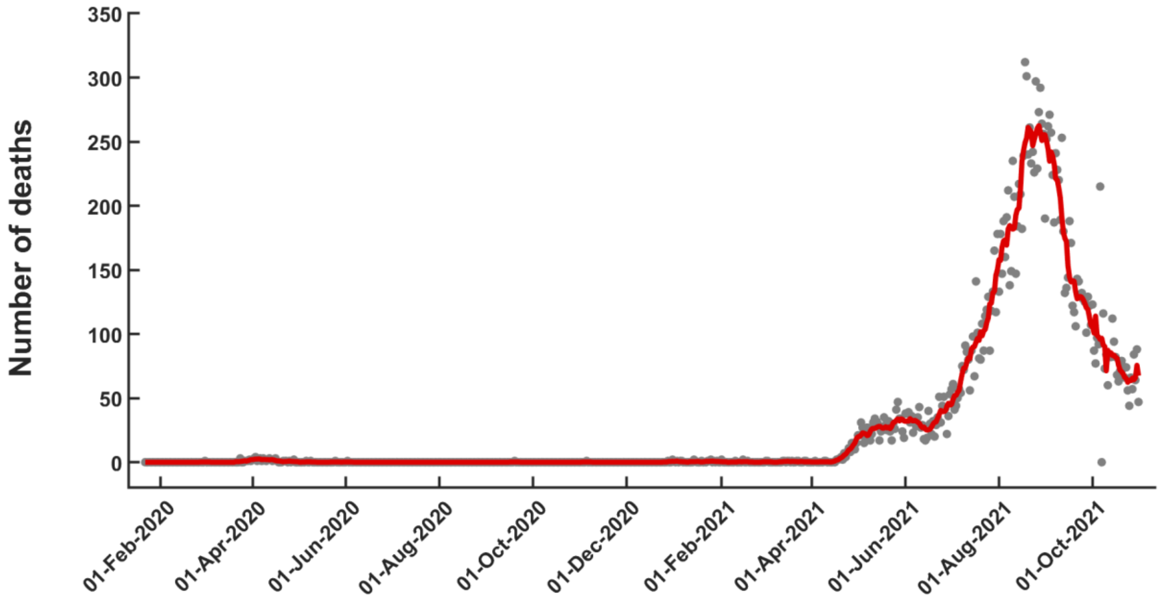

Figure S1. Daily confirmed COVID-19 deaths. The red line illustrates the 7-day average of the number of COVID-19 deaths starting from January 22, 2020, to October 31, 2021 in Thailand. Points show the observed daily mortality data.

Baseline mortality during 2015 – 2021

We used mortality data from January 2015 to December 2019 by age and sex to fit the models. For Model A, the structure of the model was described in the main text. The observed and baseline mortality of Model A stratified by age group in 2015–2021 for men and women are shown in Figure S2 and S3, respectively.

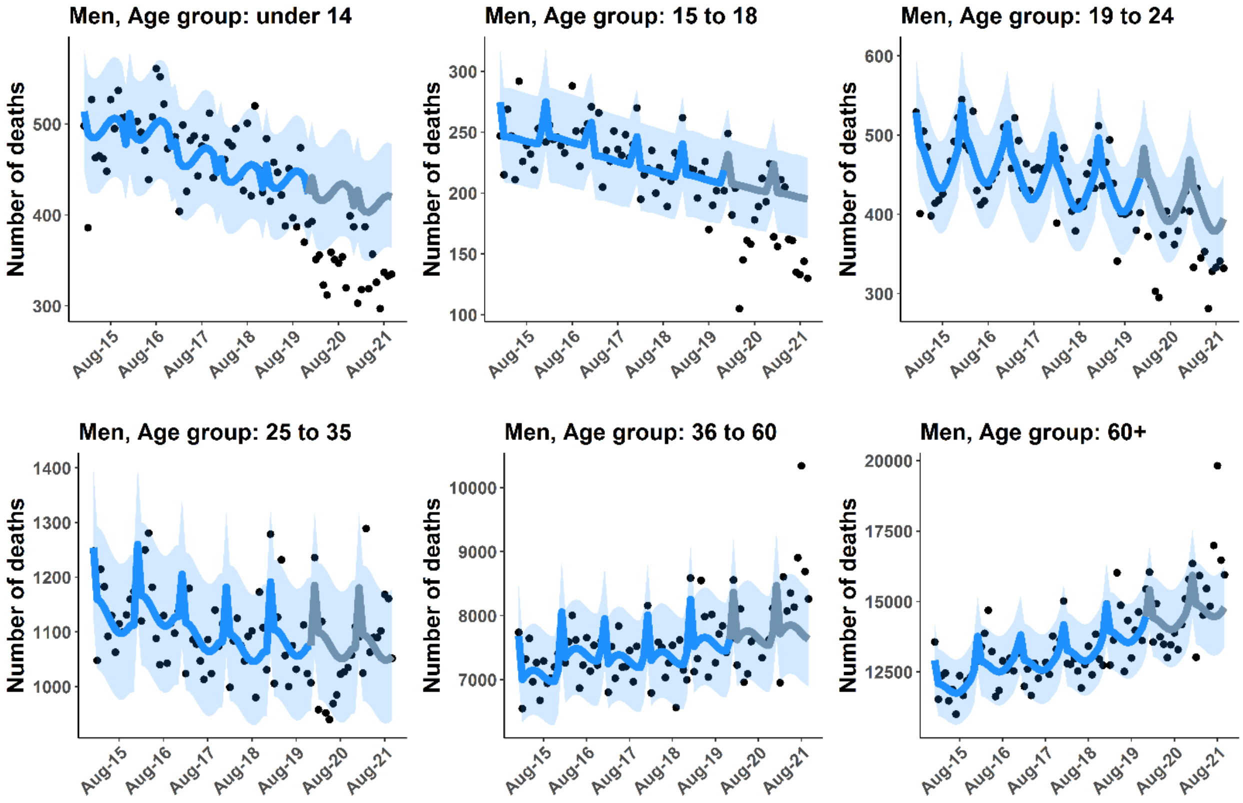

Figure S2. Men baseline mortality from Model A. Lines illustrate the baseline mortality by six age groups (0-14, 15-18, 19-24, 25-34, 35-60, and over 60 years of age). Grey lines show the predicted baseline starting from January 2020 to October 2021. Points show the observed mortality data. Shaded areas indicate 95% CI.

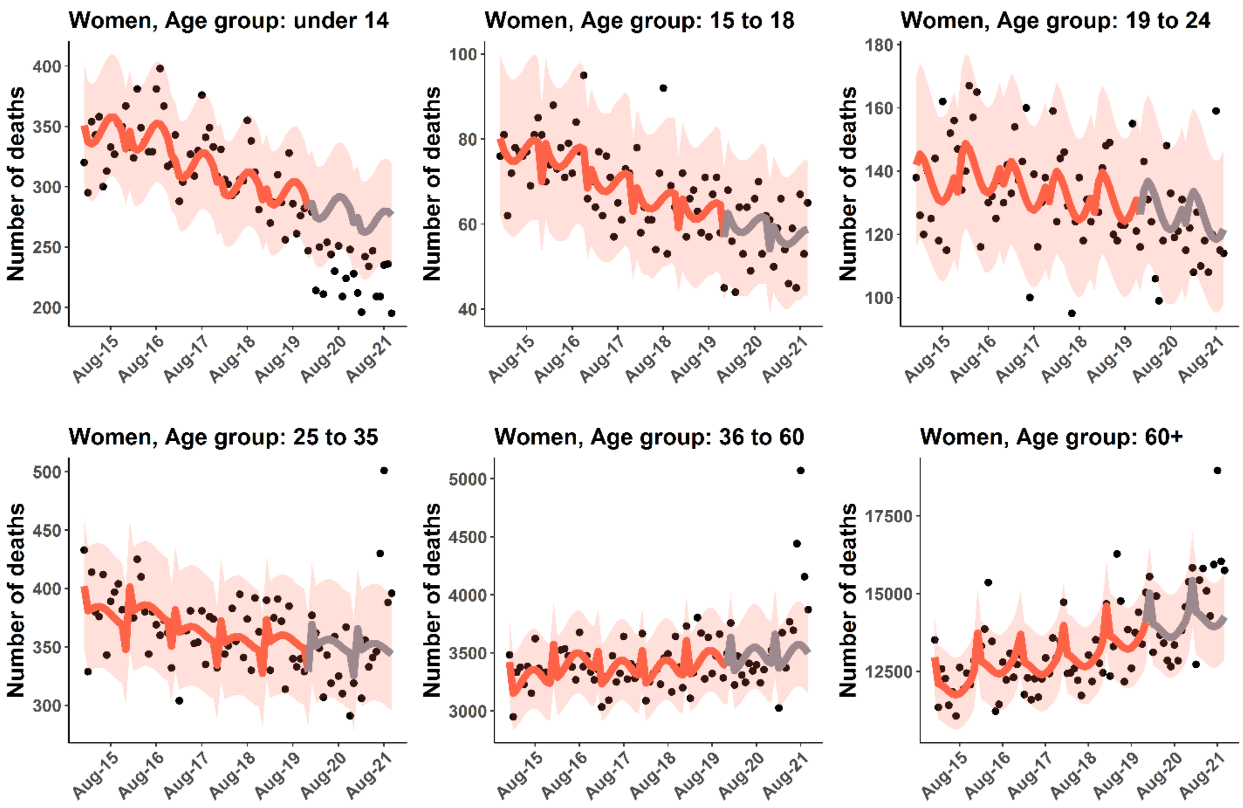

Figure S3. Women baseline mortality from Model A. Lines illustrate the baseline mortality by six age groups (0-14, 15-18, 19-24, 25-34, 35-60, and over 60 years of age). Grey lines show the predicted baseline starting from January 2020 to October 2021. Points show the observed mortality data. Shaded areas indicate 95% CI.

The other three models have different structures, as follows.

Model B:

$$Y_t \sim \beta_1 * time_t * sex_t * agegroup_t$$

$$+\beta_2 * \sin\left(\frac{2\pi * (month/30)}{365.25}\right) * sex_t * agegroup_t$$

$$+\beta_3 * \cos\left(\frac{2\pi * (month/30)}{365.25}\right) * sex_t * agegroup_t$$

$$+\beta_4 * offset(\log(exposure_t))$$

$$+\mu(1|year)$$

The observed and baseline mortality stratified by age group in 2015-2021 for men and women, are shown in Figure S4-S5, respectively.

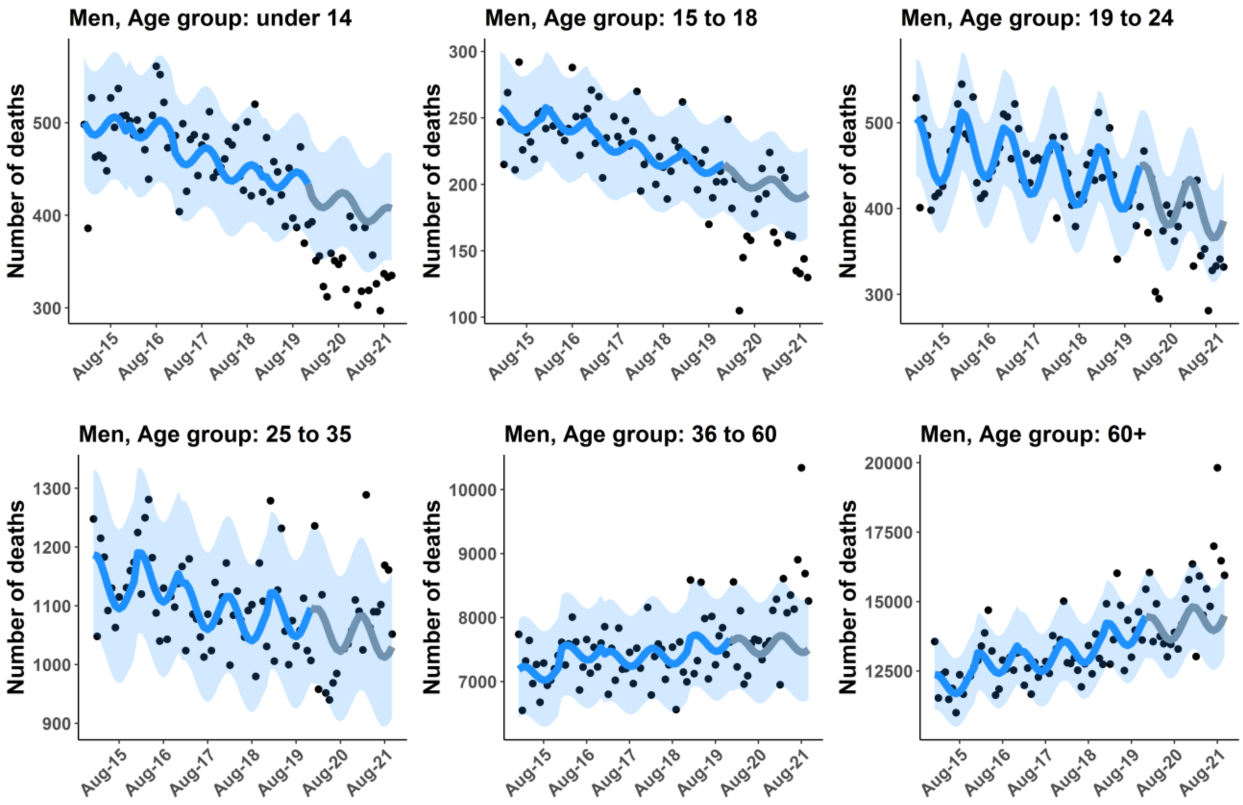

Figure S4. Men baseline mortality from Model B. Lines illustrate the baseline mortality by six age groups (0-14, 15-18, 19-24, 25-34, 35-60, and over 60 years of age). Grey lines show the predicted baseline starting from January 2020 to October 2021. Points show the observed mortality data. Shaded areas indicate 95% CI.

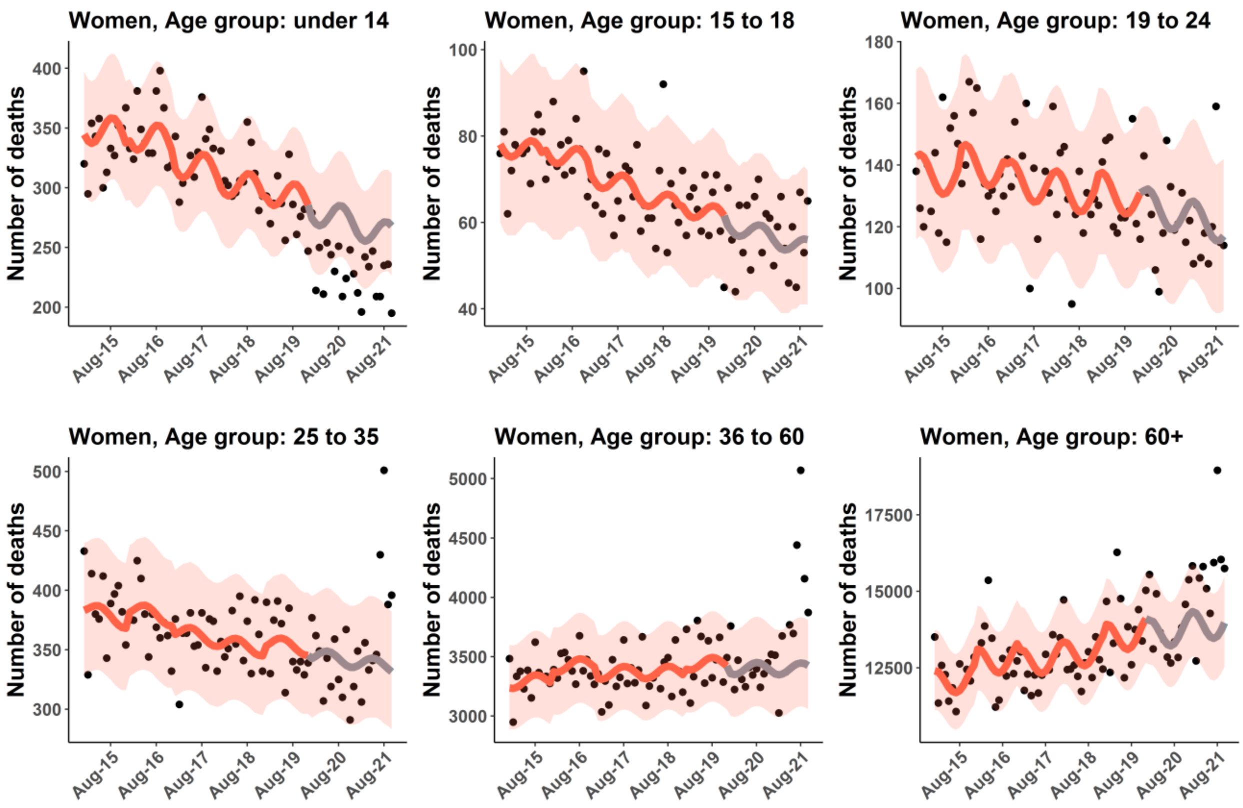

Figure S5. Women baseline mortality from Model B. Lines illustrate the baseline mortality by six age groups (0-14, 15-18, 19-24, 25-34, 35-60, and over 60 years of age). Grey lines show the predicted baseline starting from January 2020 to October 2021. Points show the observed mortality data. Shaded areas indicate 95% CI.

Model C:

$$
\begin{aligned}
Y_t \sim\ & \beta_1 * time_t * sex_t * agegroup_t \\
& + \beta_2 * \sin\left(\frac{2\pi * (month/30)}{365.25/2}\right) * sex_t * agegroup_t \\
& + \beta_3 * \cos\left(\frac{2\pi * (month/30)}{365.25/2}\right) * sex_t * agegroup_t \\
& + \beta_4 * offset(\log(exposure_t)) \\
& + \mu(1|year)
\end{aligned}
$$

The observed and baseline mortality stratified by age group in 2015-2021 for men and women, are shown in Figure S6-S7, respectively.

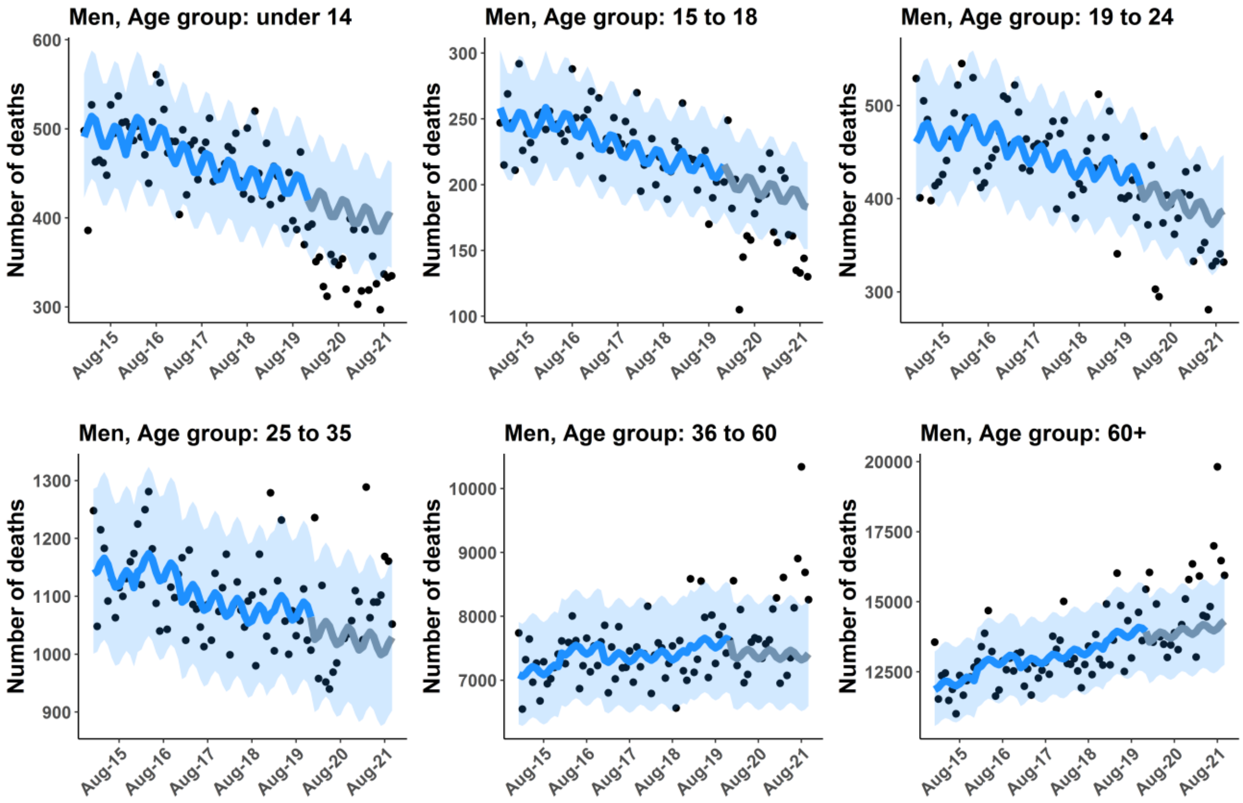

Figure S6. Men baseline mortality from Model C. Lines illustrate the baseline mortality by six age groups (0-14, 15-18, 19-24, 25-34, 35-60, and over 60 years of age). Grey lines show the predicted baseline starting from January 2020 to October 2021. Points show the observed mortality data. Shaded areas indicate 95% CI.

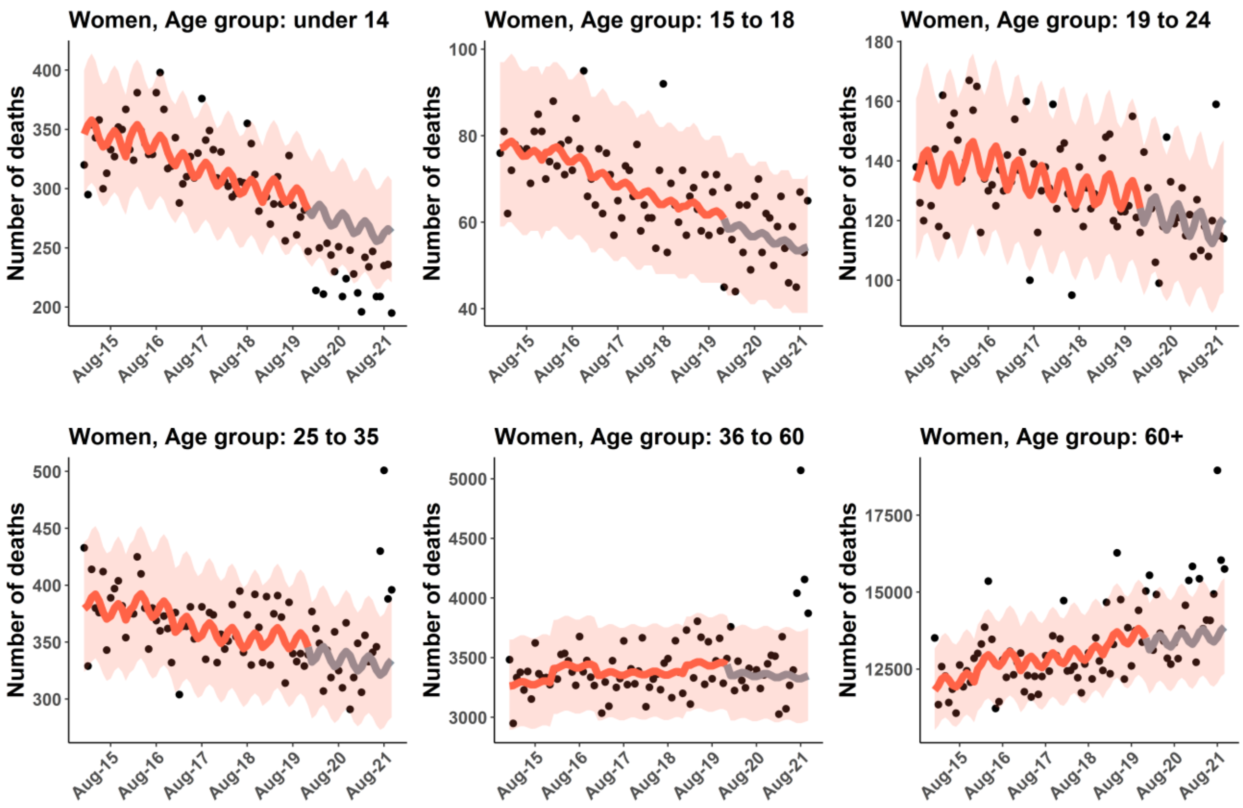

Figure S7. Women baseline mortality from Model C. Lines illustrate the baseline mortality by six age groups (0-14, 15-18, 19-24, 25-34, 35-60, and over 60 years of age). Grey lines show the predicted baseline starting from January 2020 to October 2021. Points show the observed mortality data. Shaded areas indicate 95% CI.

Model D:

$$Y_t \sim \beta_1 * time_t * sex_t * agegroup_t$$

$$+\beta_2 * \sin\left(\frac{2\pi * (month/30)}{365.25}\right) * sex_t * agegroup_t$$

$$+\beta_3 * \cos\left(\frac{2\pi * (month/30)}{365.25}\right) * sex_t * agegroup_t$$

$$+\beta_4 * \sin\left(\frac{2\pi * (month/30)}{365.25/2}\right) * sex_t * agegroup_t$$

$$+\beta_5 * \cos\left(\frac{2\pi * (month/30)}{365.25/2}\right) * sex_t * agegroup_t$$

$$+\beta_6 * offset(\log(exposure_t))$$

$$+\mu(1|year)$$

The observed and baseline mortality stratified by age group in 2015-2021 for men and women, are shown in Figure S8-S9, respectively.

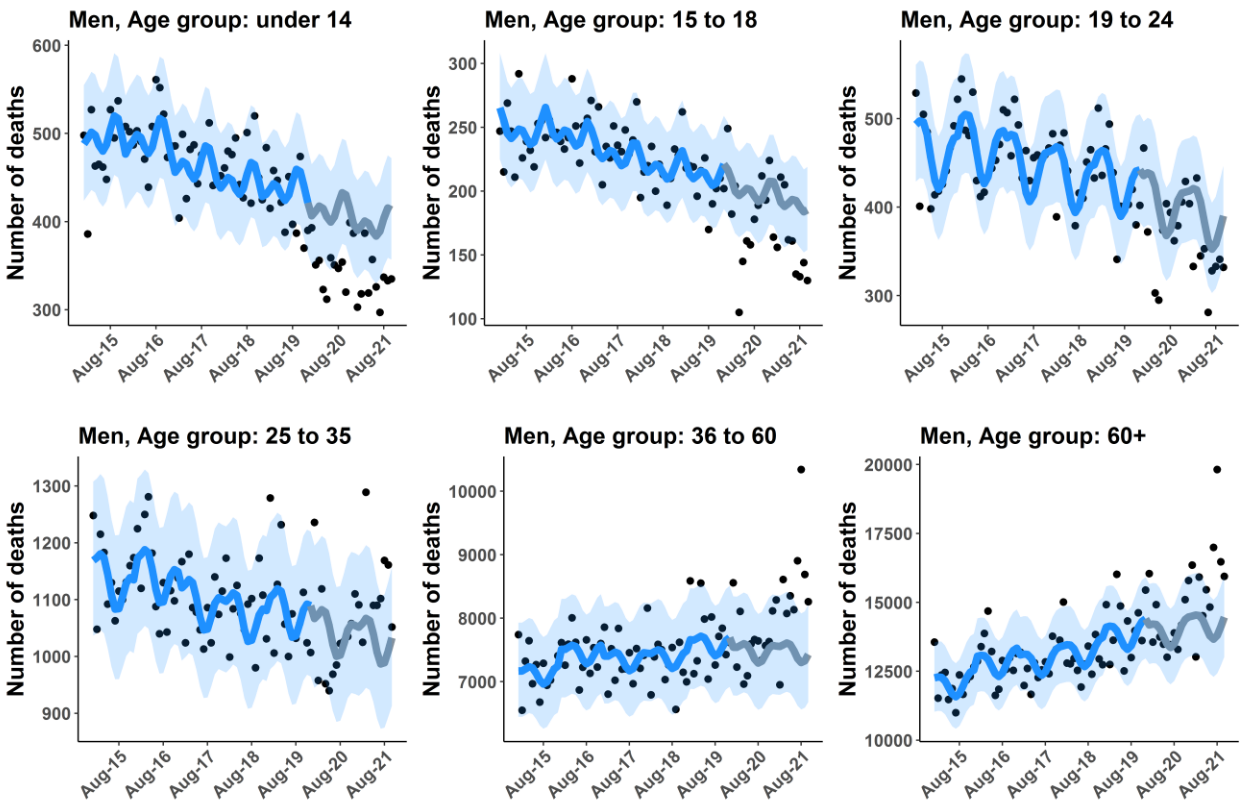

Figure S8. Men baseline mortality from Model D. Lines illustrate the baseline mortality by six age groups (0-14, 15-18, 19-24, 25-34, 35-60, and over 60 years of age). Grey lines show the predicted baseline starting from January 2020 to October 2021. Points show the observed mortality data. Shaded areas indicate 95% CI.

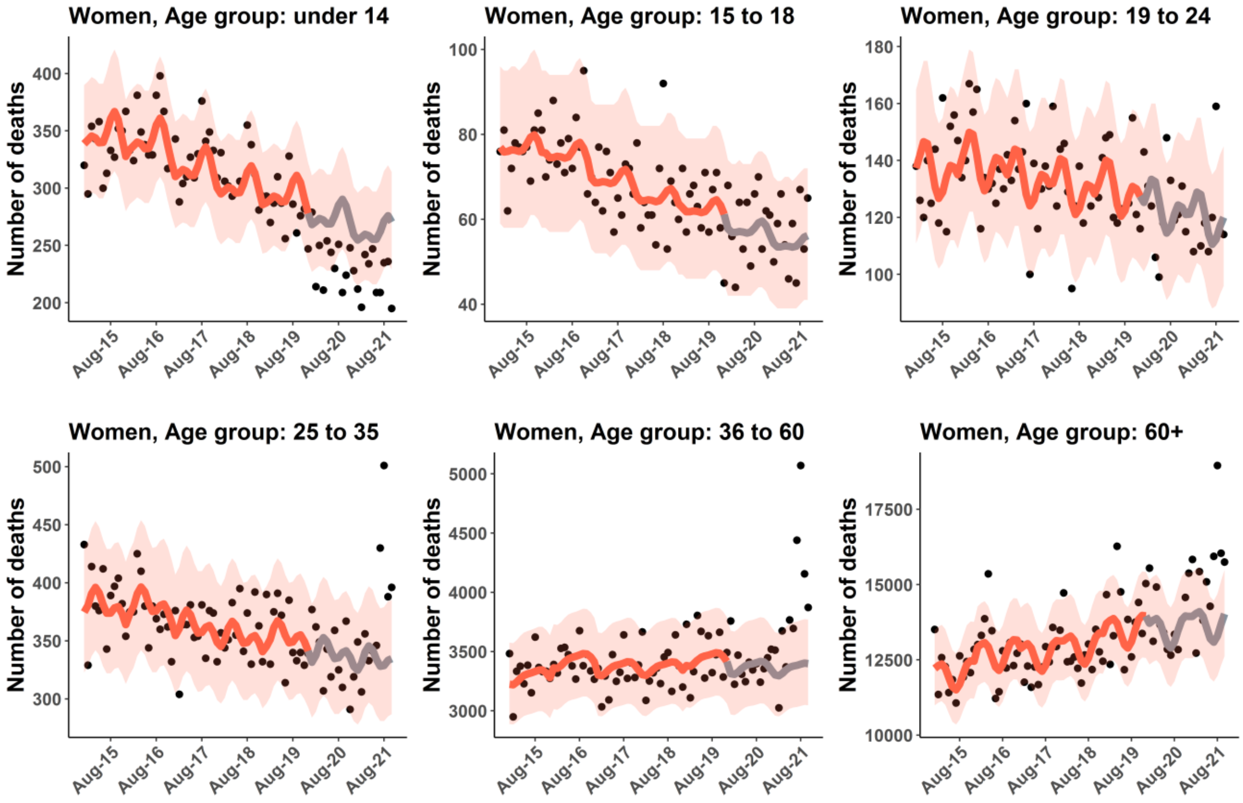

Figure S9. Women baseline mortality from Model D. Lines illustrate the baseline mortality by six age groups (0-14, 15-18, 19-24, 25-34, 35-60, and over 60 years of age). Grey lines show the predicted baseline starting from January 2020 to October 2021. Points show the observed mortality data. Shaded areas indicate 95% CI.

A summary of four different models is shown in Table S1. We used AIC to estimate the quality of each model.

Table S1. Model comparison.

Model	AIC	Number parameters	Number coefficients
Model A	8095.2	8	72
Model B	8108.1	6	48
Model C	8170.7	6	48
Model D	8123.5	8	72

Life expectancy

We used life expectancy at birth as a summary indicator of population health in Thailand. We estimated the life expectancy at birth in Thailand for the periods 2015–2021 using a life table. The life expectancy at birth of the population in Thailand is shown in Figure S10.

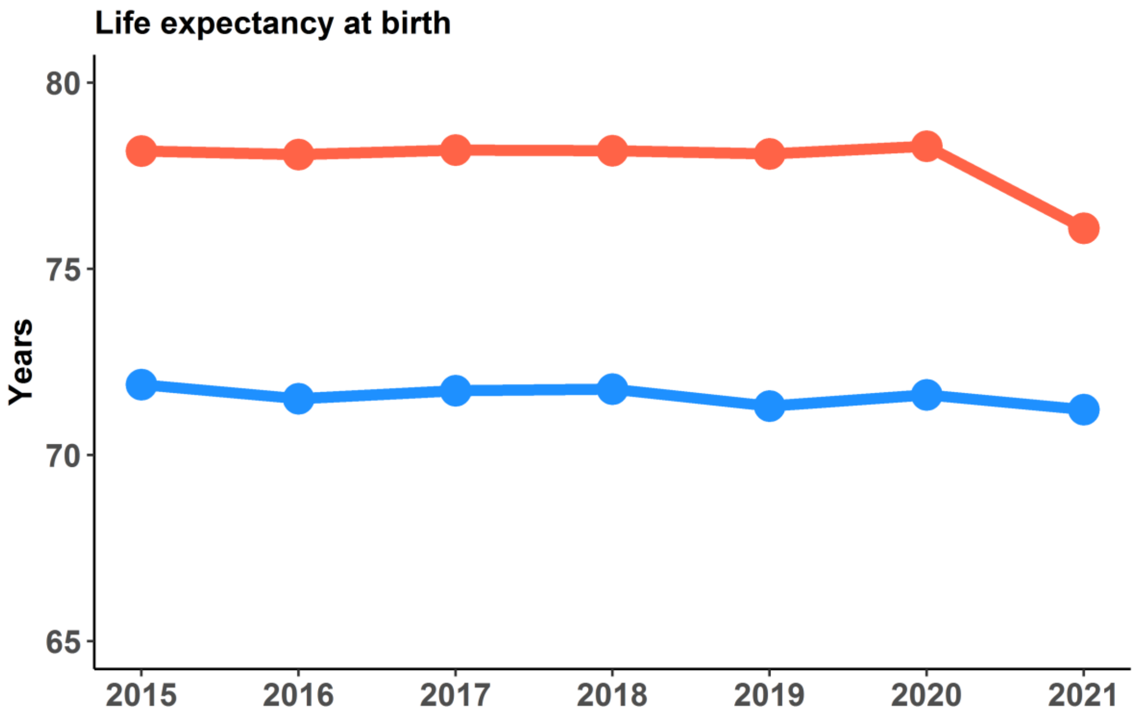

Figure S10. Life expectancy at birth. Lines illustrate the life expectancy at birth estimates for the periods 2015–2021. The blue line shows the estimated life expectancy at birth of men. The orange line shows the estimated life expectancy at birth of women in Thailand.

Pneumonia excess mortality

We used the pneumonia mortality data from January 2015 to December 2019 to estimate the baseline pneumonia mortality in the absence of COVID-19. The monthly pneumonia mortality data in Thailand was obtained from the Bureau of Epidemiology, Department of Disease Control, MoPH, Thailand [2]. The mortality data was fitted by generalized linear mixed models (GLMMs) as follows:

$$Y_t \sim \beta_1 * time_t$$

$$+\beta_2 * \sin\left(\frac{2\pi * (month/30)}{365.25}\right)$$

$$+\beta_3 * \cos\left(\frac{2\pi * (month/30)}{365.25}\right)$$

$$+\beta_4 * offset(\log(exposure_t))$$

$$+\mu(1|year).$$

We then projected the pneumonia baseline mortality forward from January 2020 to October 2021. The observed and baseline mortality of pneumonia in Thailand is illustrated in Figure S11. The pneumonia excess mortality was then calculated from the number of pneumonia deaths minus the baseline prediction.

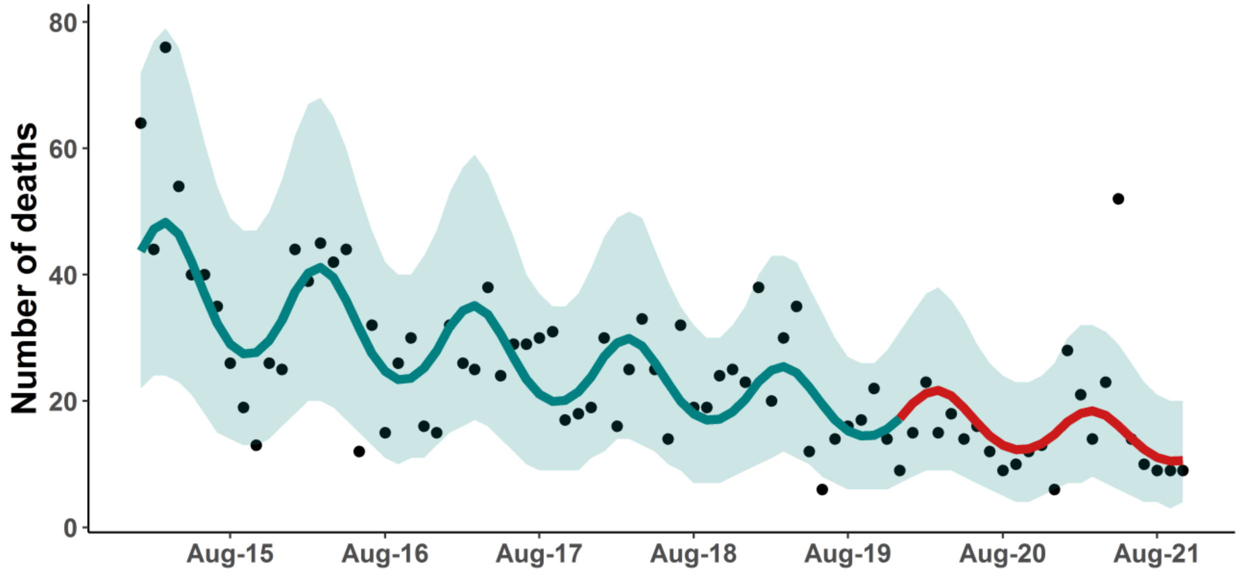

Figure S11. Pneumonia baseline mortality. Line illustrates the pneumonia baseline mortality in Thailand starting from January 2015 to October 2021. The red line shows the predicted pneumonia baseline mortality starting from January 2020 to October 2021. Points show the observed pneumonia mortality data. The shaded area indicates 95% CI.

Traffic accident mortality data

Figure S12 shows the traffic accident mortality deaths from January 2017 to October 2021 and the mobility trends in Thailand. Data on traffic accident-related deaths was acquired from the Road Accidents Data Center for Road Safety Culture of Thailand (Thai RSC) [3]. The accident mortality data were also stratified by age group and gender (Figure S13**).** The number of people dying from traffic accidents was categorized into six age groups (0-14, 15-18, 19-24, 25-34, 35-60, and over 60 years of age). The data suggested that after the Thai government implemented social distancing and stringent lockdown measures, the number of traffic accident deaths decreased by approximately 20% compared with the average traffic accident deaths of the previous year. Moreover, we used the mobility in Thailand reported by Apple [4] as a representative of the mobility in Thailand during the lockdown. Driving and walking mobility tend to decrease compared with baseline.

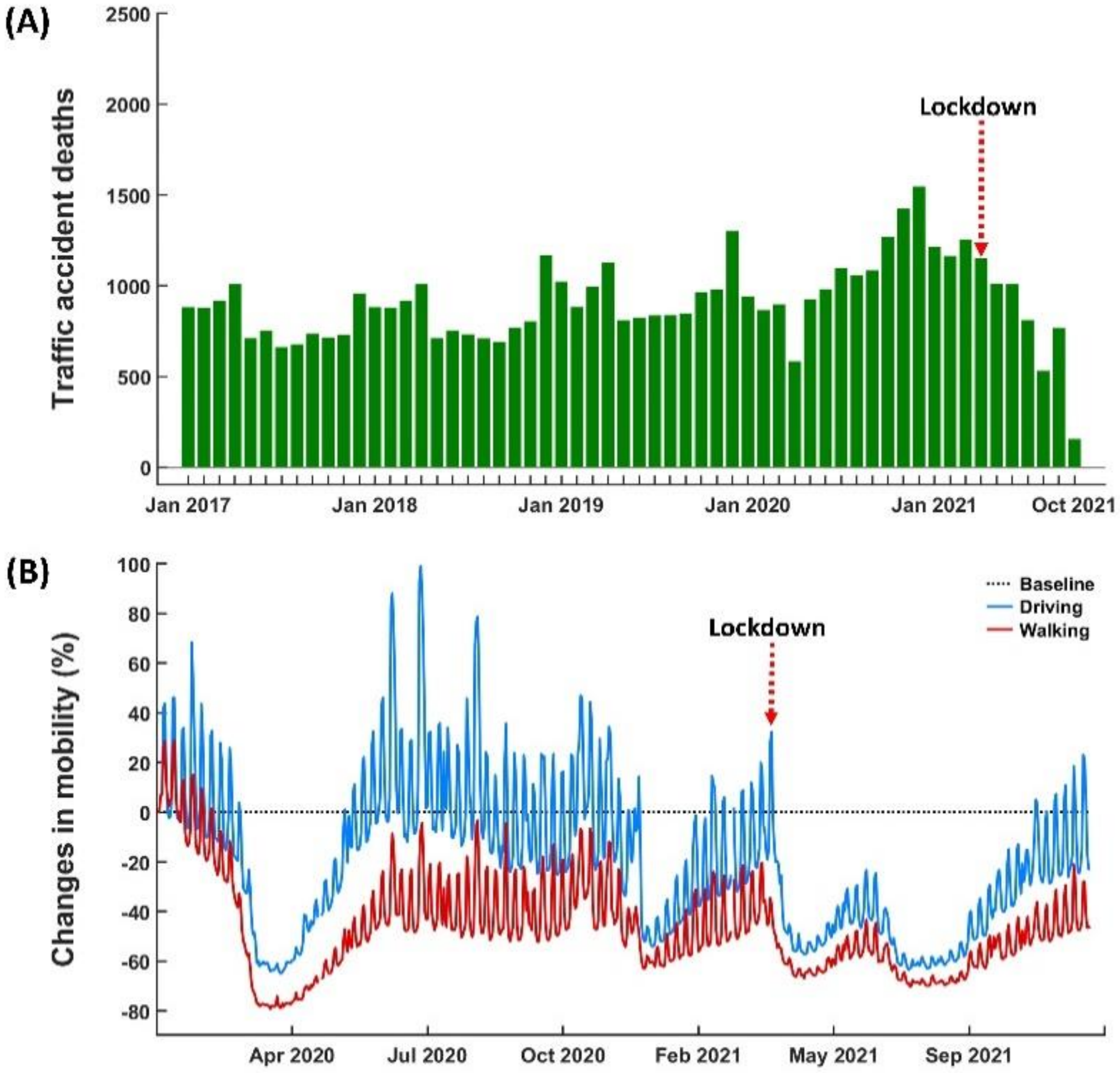

Figure S12. Traffic accident mortality data and mobility trends data. (A) Traffic accident mortality data from January 2017 to October 2021. (B) The mobility in Thailand reported by Apple.

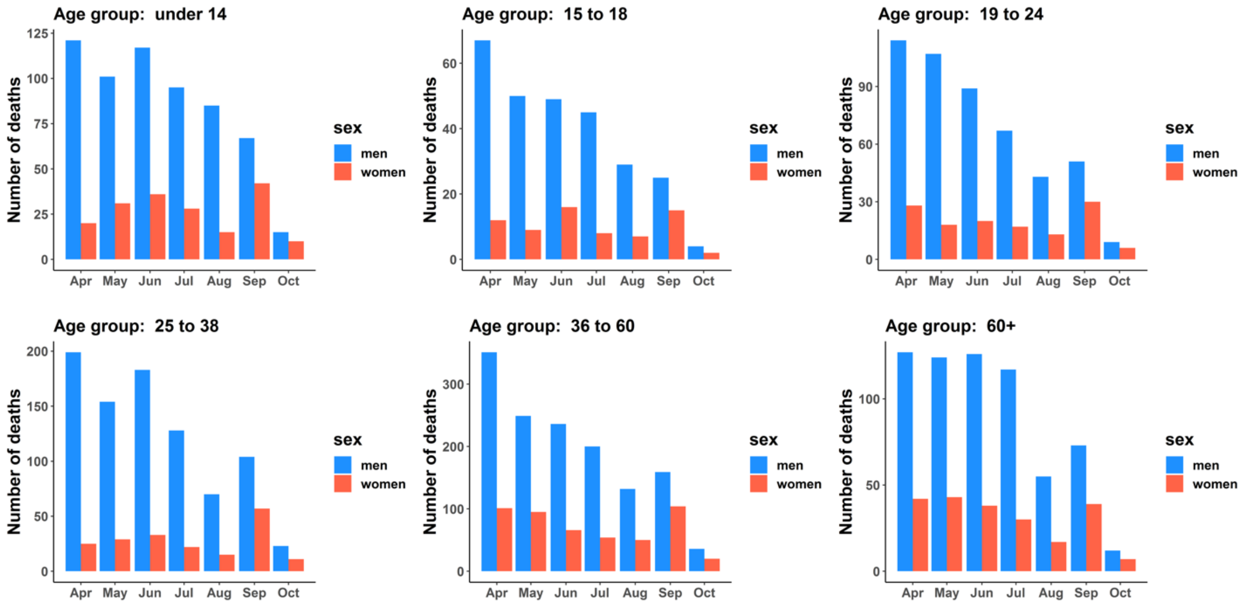

Figure S13. Traffic accident deaths by gender and age groups. Bars show the monthly traffic accident deaths by gender and age groups starting from April to October 2021.

References

1. Johns Hopkins University. *Dashboard by the Center for Systems Science and Engineering (CSSE) at Johns Hopkins University (JHU)*; John Hopkins University Coronvirus Resource Centre: Baltimore, MD, USA, 2020.
2. Bureau of Epidemiology, Department of Disease Control, MoPH, Thailand. Pneumonia. Available online: http://doe.moph.go.th/surdata/disease.php?dcontent=old&ds=31 (accessed on 4 January 2022).
3. Road Accidents Data Center for Road Safety Culture of Thailand, T. Traffic Accident-Related Mortality Data. Available online: https://www.thairsc.com/ (accessed on 30 September 2021).
4. Apple. COVID-19—Mobility Trends Reports Apple. 2021. Available from: https://covid19.apple.com/mobility.(accessed on 3 November 2021).
